# Association between subjective symptoms and obesity and postoperative recurrence in differentiated thyroid cancer: a matched-pair analysis

**DOI:** 10.1038/s41598-020-60276-z

**Published:** 2020-02-28

**Authors:** Yu-Ying Wu, Shih-Ping Cheng, Piao-Yi Chiou, Chieh-Yu Liu

**Affiliations:** 1 Department of Surgery, MacKay Memorial Hospital, Taipei, 10449 Taiwan (R.O.C.); 2 Department of Surgery, MacKay Memorial Hospital and Mackay Medical College, Taipei, 10449 Taiwan (R.O.C.); 3 School of Nursing, Medical College, National Taiwan University; Department of Nursing, National Taiwan University Hospital, Taipei, 10055 Taiwan (R.O.C.); 4 Biostatistical Consultant Lab, Department of Speech Language Pathology and Audiology, National Taipei University of Nursing and Health Sciences, Taipei, 11219 Taiwan (R.O.C.)

**Keywords:** Thyroid cancer, Surgical oncology

## Abstract

Several patients with thyroid cancer experience symptom distress after diagnosis and surgery. Data on the association between symptomatology and disease recurrence are limited. A retrospective review of a prospectively maintained database was performed, and 57 patients who had recurrence after operation of differentiated thyroid cancer were identified. Controlling for age, sex, surgery, and tumour stage, 114 patients without recurrent disease were selected by case-control matching. Subjective symptoms at follow-up visits were extracted from medical records and classified into three symptom clusters: pharyngolaryngeal, psychoneurological, and gastrointestinal. Compared to the control group, patients with recurrence had higher symptom totals in the pharyngolaryngeal (*P* < 0.001) and psychoneurological clusters (*P* = 0.005). Symptom score >3 yielded a sensitivity of 61.4% and a specificity of 80.7% to predict recurrence. Multivariate Cox regression analysis revealed that high symptom score (hazard ratio [HR] = 4.184), family history of thyroid cancer (HR = 2.463), and obesity (HR = 1.981) were independently associated with disease recurrence. Taken together, the likelihood of postoperative recurrence seemed to increase with high self-perceived symptom burden, family history, and obesity in patients with thyroid cancer. The results could be applied to the recurrence surveillance and symptomatic management of thyroid cancer post-operation.

## Introduction

A steady increase in the incidence of differentiated thyroid cancer has been observed worldwide^1^. In recent years, the trend appears to be stabilised as a result of awareness of overdiagnosis and overtreatment of thyroid cancer^2^. After the initial therapy, which consists of thyroidectomy with or without radioiodine ablation, postoperative surveillance is usually performed. The rationale for surveillance is based on the existing evidence that persistent or recurrent events are common even decades after the establishment of diagnosis, although most thyroid cancers have an excellent prognosis after appropriate treatment^3,4^. The risk of persistent or recurrent disease is primarily determined by tumour biology. Age, sex, and a few clinicopathological factors such as tumour size, nodal metastasis, extrathyroidal extension, and distant metastasis have been shown to influence the risk of recurrence^4,5^. Notably, there is a significant overlap between the risk factors for recurrence and predictors of death^4^.

The economic burden of continuing surveillance of patients with thyroid cancer who have a long life expectancy could be substantial^6^. Efforts have been made to establish an individual’s risk profile and guide surveillance intensity and duration to promote the efficacy of the surveillance. It is increasingly recognised that several low-risk patients with excellent response to therapy have a significantly low incidence of recurrence, and a simple assessment schedule may be sufficient^7^. As such, the implementation of dynamic risk stratification has considerably refined the initial estimates of recurrence risk. Dynamic risk stratification is mainly based on biochemical and imaging data collected during follow-up. The current risk scheme of thyroid cancer has insufficient input of clinical symptomatology and assessment by the healthcare givers.

Large cohort studies have demonstrated that patients with thyroid cancer experience symptom distress frequently after diagnosis, which negatively affects their quality of life^8–10^. Furthermore, thyroid cancer survivors reported worse quality of life in the domains of anxiety, depression, fatigue, and sleep disturbance than individuals with other cancers^11^. In some cancer types, patient-perceived symptoms are the most frequent indicators of recurrence^12,13^. However, it remains unknown whether subjective symptoms are associated with thyroid cancer recurrence. Additionally, some controversies regarding the association between obesity and aggressive features of thyroid cancer exist^14,15^. It will be interesting to investigate the association between obesity and patient symptoms.

In the present study, we aimed to evaluate whether subjective symptoms and obesity are associated with disease recurrence of thyroid cancer. Hence, we performed a matched-pair analysis to compare self-reported symptoms and body mass index (BMI) between patients with and without recurrence.

## Methods

### Patients and data collection

We queried our prospectively maintained database from a medical center to retrospectively review and identify eligible patients. All data collection and analysis were performed after the study was approved by the Institutional Review Board of MacKay Memorial Hospital (18MMHIS154), which waived the need for patient consent. All identifiable profiles of each patient were unlinked to protect the patients privacy. The inclusion criterion for this study was as follows: adult patients who underwent lobectomy or total thyroidectomy for differentiated thyroid cancer from 2001 to 2017. The exclusion criteria included the following: patients undergoing biopsy only, the index operation performed elsewhere, age younger than 20 years at diagnosis, second primary malignancy other than thyroid cancer, and short follow-up (<6 months). Patient demographics, comorbidities, tumour histology, and laboratory and imaging results were obtained from inpatient, outpatient, and procedure records.

### Treatment and follow-up

Total thyroidectomy was performed at our institution when the tumour met either of the following criteria: (1) maximum tumour size >4 cm, (2) gross extrathyroidal extension, (3) clinically apparent lymph node metastasis, or (4) presence of distant metastasis^16^. Other patients were treated with either lobectomy or total thyroidectomy per operating surgeon’s discretion. The decision for radioactive iodine therapy and the prescribed radioiodine activity were determined based on available clinical and histopathological information.

Patients were usually assessed and examined every 3 to 6 months. During the follow-up visits, all patients underwent ultrasound examination of the neck and thyroglobulin (Tg) and anti-Tg antibody measurements^17^. The follow-up period for each patient was defined as the length of time from the initial therapy until the last known contact documented by the medical record. The database was closed in December 2018, and data were double verified before the analysis started.

When any evidence of disease was present within 6 months following initial treatment, the patient was considered to have persistent disease. Recurrence was defined by structural or functional evidence of disease after a disease-free status of 6 months or longer regardless of the Tg levels with or without anti-Tg antibodies. Regarding locoregional recurrence, the diagnosis was based on cytological or histopathological confirmation. Distant recurrence was typically determined by imaging studies, including radioiodine scans, computed tomography, positron emission tomography, or cytological/histopathological evidence when available. Biochemical alterations were not considered as an indicator of recurrence in this study.

### Matching procedure

A total of 1099 patients met the inclusion criteria during the period of data collection. Among them, 57 had postoperative recurrence and were designated as the case group. Each patient with disease recurrence was matched to a non-recurrent patient for age, sex, surgical therapy, and disease stage according to tumor-node-metastasis (TNM) classification. Case-control matching was performed using the fuzzy extension in Python Essentials, International Business Machines Corporation Statistical Package for the Social Sciences (IBM SPSS) Statistics. Recurrent and non-recurrent cases were matched in a 1:2 fashion to increase statistical power without risking bias^18^. A fuzzy matching algorithm was used to ensure that there was no difference in the matching criteria between the groups. After matching for age, sex, surgery, and TNM stage, 114 patients without persistent disease were selected as the control cohort for this study. These 171 patients constituted the matched study cohort.

### Symptom clusters and obesity definition

Physical and psychological symptoms were recorded during the systematic review routinely used in medical history obtained at follow-up visits. These subjective symptoms were retrospectively derived from the hospital charts. Considering that multiple symptoms may offer higher clinical significance than individual symptoms, common symptoms were further classified into three symptom clusters^19^. The pharyngolaryngeal cluster consisted of globus sensation, sore throat, sputum retention, frequent throat clearing or chronic cough, and voice change. The psychoneurological cluster consisted of palpitation, anxiety, sleep disturbance, fatigue, numbness, muscle twitches and spasms, cold sweats, dizziness, and headache. The gastrointestinal cluster consisted of body weight loss, weight gain, upset stomach, lack of appetite, and taste alterations. Each symptom was graded as present or absent irrespective of the frequency or duration of symptoms. A total symptom score was calculated by adding up all symptoms across the three clusters.

Patients’ body weight was recorded at initial diagnosis. The definition of obesity was based on the cutoff values suggested by the Ministry of Health and Welfare, Taiwan^20^. Underweight was defined as BMI < 18.5 kg/m^2^, overweight as BMI between 24 and 27 kg/m^2^, and obesity as BMI ≥ 27 kg/m^2^.

### Statistical analysis

The primary outcome of this study was to examine the association between symptom clusters and BMI and recurrence among patients with differentiated thyroid cancer. Categorical variables were expressed as frequencies and percentages, and continuous variables were expressed as mean ± standard deviation. Categorical variables were evaluated with the chi-squared test or Fisher’s exact test as appropriate. The comparisons between groups were performed using the unpaired Student’s *t-*test for continuous variables. A receiving operating characteristic (ROC) curve analysis was performed to identify the sensitivity and specificity of a cutoff value of symptom scores to predict the recurrence of differentiated thyroid cancer. Disease-specific survival curves were plotted using the Kaplan-Meier method, and the log-rank test was used to assess the statistical significance between the curves. A Cox proportional hazard regression model was used to identify potential predictive factors for recurrence. Any variable with a *P* value < 0.1 in the univariate analysis was subsequently included in the multivariable model. Hazard ratio (HR) and 95% confidence intervals (CIs) were calculated for each variable. All *P* values were two-tailed, and *P* < 0.05 was considered statistically significant. All analyses were performed using the IBM SPSS Statistics for Windows version 22.0 (IBM Corporation, Armonk, NY, USA).

## Results

### Patient characteristics

A total of 1099 adult patients underwent surgical treatment for differentiated thyroid cancer at our institution from 2001 to 2017. At the end of 2018, recurrence was documented in 57 (5.2%) patients, including locoregional recurrence in 39 patients, contralateral thyroid recurrence in 4 patients, lung metastasis in 6 patients, bone metastasis in 2 patients, locoregional and lung recurrence in 5 patients, and concurrent lung and bone metastasis in 1 patient. The mean time to recurrence was 5.9 ± 4.3 years (median, 5.0 years).

The control group consisted of 114 patients without persistent disease who were selected using fuzzy case-control matching. The demographic characteristics of the 171 patients are shown in Table 1. The mean age was 48.5 ± 14.2 years, and most of the patients were female (n = 127, 74%). Approximately 10% of the patients had a family history of thyroid cancer.

**Table 1:** Characteristics of 171 patients with differentiated thyroid cancer in this study.

Variables	All (n = 171)	No recurrence (n = 114)	Recurrence (n = 57)	*P* value
Age (years)	48.5 ± 14.2	48.3 ± 13.5	48.9 ± 15.7	0.802
Sex				0.902
Male	44 (26%)	29 (25%)	15 (26%)	
Female	127 (74%)	85 (75%)	42 (74%)	
Body mass index (kg/m^2^)	24.3 ± 3.8	24.0 ± 3.7	24.9 ± 4.0	0.146
BMI category				0.049
Underweight	5 (3%)	2 (2%)	3 (5%)	
Normal	86(50%)	65 (57%)	21 (37%)	
Overweight	42 (25%)	26 (23%)	16 (28%)	
Obesity	38 (22%)	21 (18%)	17 (30%)	
TNM stage				0.605
Stage I	82 (48%)	56 (49%)	26 (46%)	
Stage II	7 (4%)	3 (3%)	4 (7%)	
Stage III	28 (16%)	19 (17%)	9 (16%)	
Stage IV	54 (32%)	36 (32%)	18 (32%)	
Hypertension				0.411
No	119 (70%)	77 (68%)	42 (74%)	
Yes	52 (30%)	37 (32%)	15 (26%)	
Diabetes mellitus				0.874
No	148 (87%)	99 (87%)	49 (86%)	
Yes	23 (13%)	15 (13%)	8 (14%)	
Hyperlipidemia				0.599
No	134 (78%)	88 (77%)	46 (81%)	
Yes	37 (22%)	26 (23%)	11 (19%)	
Family history				0.206
No	154 (90%)	105 (92%)	49 (86%)	
Yes	17 (10%)	9 (8%)	8 (14%)	
Total symptom score	3.0 ± 1.7	2.4 ± 1.4	4.0 ± 1.7	<0.001
Pharyngolaryngeal	1.3 ± 1.2	1.1 ± 1.0	1.9 ± 1.3	<0.001
Psychoneurological	1.3 ± 1.2	1.1 ± 1.1	1.7 ± 1.3	0.005
Gastrointestinal	0.2 ± 0.5	0.2 ± 0.4	0.3 ± 0.6	0.264

Abbreviations: BMI, body mass index; TNM, tumor-node-metastasis.

### Features of the recurrence group

**Figure 1:**
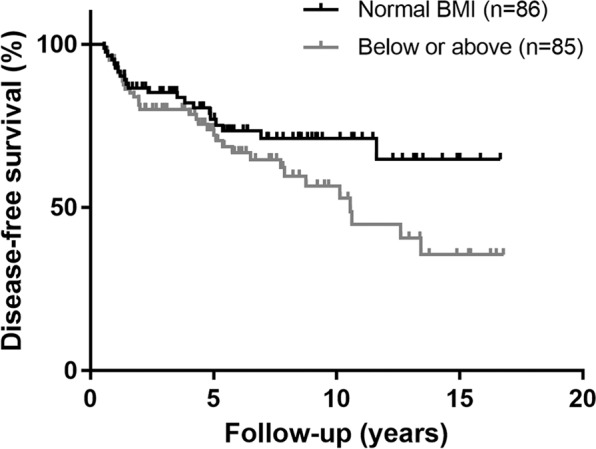
Kaplan-Meier plot of disease-free survival of the matched cohort with differentiated thyroid cancer, stratified by body mass index (BMI).

As expected, age, sex, and disease stage were comparable between the 57 patients with recurrence and 114 patients in the control group. The prevalence of common comorbidities, including hypertension, diabetes mellitus, and hyperlipidaemia, was similar between the two groups (Table 1). Interestingly, patients with recurrence were less likely to have a normal weight (*P* = 0.049). Consistently, we found that patients with a normal weight had longer disease-free survival than those who were underweight, overweight, or obese (log-rank test *P* = 0.040, Fig. 1).

### Symptom profile

**Figure 2:**
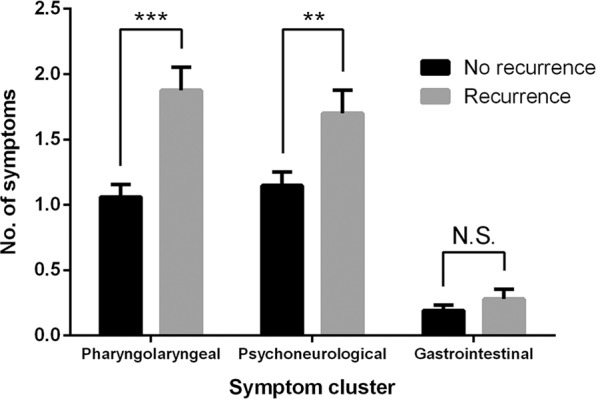
Symptom scores stratified by three clusters in patients with differentiated thyroid cancer. **P < 0.01; ***P < 0.001; N.S., non-significant.

Symptoms in the pharyngolaryngeal and psychoneurological clusters were common in patients with thyroid cancer during follow-up. Only 26% and 29% of the patients were free of any symptoms in the pharyngolaryngeal and psychoneurological clusters, respectively. The presence of symptoms in the pharyngolaryngeal cluster was closely associated with the presence of symptoms in the psychoneurological cluster (*P* = 0.060). On the contrary, the majority (81%) of patients had no symptoms in the gastrointestinal cluster. As shown in Fig. 2, patients with disease recurrence had more symptoms in the pharyngolaryngeal and psychoneurological clusters (*P* < 0.001 and *P* = 0.005, respectively) than patients without disease recurrence. There was no difference in the symptom score of the gastrointestinal cluster.

**Figure 3:**
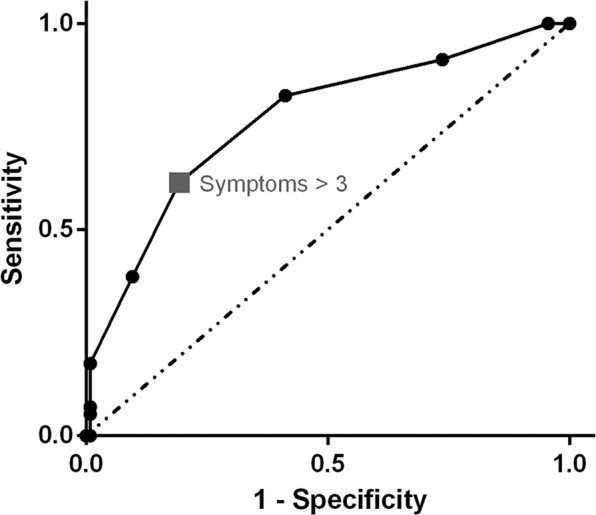
Receiving operating characteristic (ROC) analysis of the accuracy for symptom scores in the prediction of recurrence in differentiated thyroid cancer.

**Figure 4:**
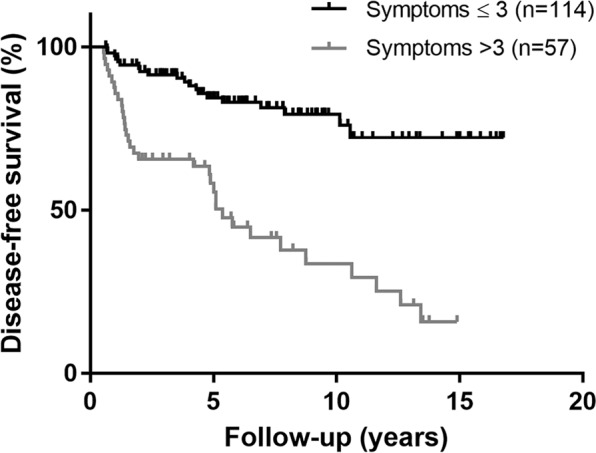
Kaplan-Meier plot of disease-free survival of the matched cohort with differentiated thyroid cancer, stratified by symptom scores.

The mean symptom total was 3.0 ± 1.7 (median, 3; range, 0 to 9). Patients with recurrence had a higher total symptom score than those without recurrence (4.0 ± 1.7 versus 2.4 ± 1.4, *P* < 0.001). We performed a ROC curve analysis to evaluate the overall predictive accuracy of the total symptom score in disease recurrence. The area under the ROC curve was 0.766 (95% CI, 0.690 to 0.842). An optimal cutoff value was determined according to the Youden index. Accordingly, a symptom score higher than 3 yielded a sensitivity of 61.4% and a specificity of 80.7% (Fig. 3). Patients who had a symptom score >3 had a significantly shorter disease-free interval than patients who had a symptom score <3 (log-rank test *P* < 0.001, Fig. 4), indicating that recurrence was associated with heightened symptom burden.

### Multivariate Cox regression analysis

**Figure 5:**
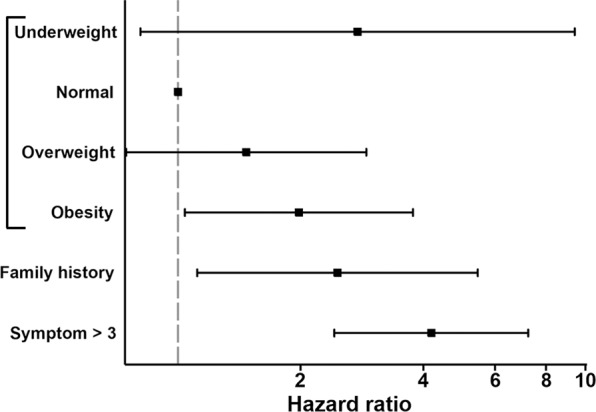
Forest plot for hazard ratio and 95% confidence intervals for the association of variables with disease-free survival in differentiated thyroid cancer. The vertical dashed line indicates hazard ratio = 1.

The association between symptom score and obesity on thyroid cancer recurrence was subsequently assessed using Cox regression analysis. During a mean follow-up of 5.9 ± 4.3 years, the multivariable-adjusted HRs for recurrence were calculated. According to our predefined criteria, BMI category (categorical), family history of thyroid cancer (binary), and total symptom score (binary), which achieved or were close to statistical significance (*P* < 0.1), were subsequently included in a multivariate analysis (Table 2). On multivariate analysis, obesity (HR = 1.981, *P* = 0.038), family history (HR = 2.463, *P* = 0.026), and high symptom score (HR = 4.184, *P* < 0.001) remained independently associated with disease recurrence. A forest plot for hazard ratios is shown in Fig. 5.

**Table 2:** Univariate and multivariate Cox regression analysis of variables associated with recurrence in differentiated thyroid cancer.

Variables	Univariate analysis			Multivariate analysis		
	Hazard ratio	95% Confidence interval	*P* value	Hazard ratio	95% Confidence interval	*P* value
Sex (female)	1.034	0.573–1.866	0.912			
Age^a^	1.005	0.987–1.024	0.564			
BMI category						
Underweight	2.650	0.789–8.902	0.115	2.758	0.809–9.408	0.105
Normal	1			1		
Overweight	1.617	0.844–3.100	0.148	1.471	0.747–2.899	0.265
Obesity	1.775	0.936–3.366	0.079	1.981	1.040–3.773	0.038
TNM stage^a^	1.104	0.910–1.339	0.317			
Hypertension	0.733	0.406–1.323	0.303			
Diabetes mellitus	1.018	0.482–2.151	0.963			
Hyperlipidemia	0.783	0.405–1.514	0.467			
Family history	2.050	0.961–4.373	0.063	2.463	1.115–5.441	0.026
Symptom score >3	4.027	2.357–6.881	<0.001	4.184	2.419–7.234	<0.001

^a^Included in the model as a continuous variable. Abbreviations: BMI, body mass index; TNM, tumor-node-metastasis.

## Discussion

In this study, we performed a matched-pair analysis to control for classical prognostic factors (age, sex, surgery, and TNM stage) for thyroid cancer. Considering that recurrence is less common than persistent disease in thyroid cancer^3^, a direct multivariate analysis incorporating these classical prognostic factors may mask the associations with other minor determinants. With our approach incorporating both case-control matching and multivariate models, we found that high subjective symptom burden, a positive family history of thyroid cancer, and obesity were independently associated with recurrence of differentiated thyroid cancer.

Subjective symptoms following the treatment of thyroid cancer are frequently reported^8,9^. The most commonly used prospective assessment instrument is the MD Anderson Symptom Inventory for thyroid cancer (MDASI-Thy)^21^. The MDASI-Thy questionnaire contained 13 core symptoms and 6 interference items and 6 symptoms specific to thyroid cancer (hoarseness, feeling hot, feeling cold, racing heartbeat, difficulty swallowing, and diarrhoea). In this study, our patient-perceived symptoms had a significant overlap with the MDASI-Thy items. Nonetheless, we found that some symptoms such as feeling hot/cold and diarrhoea were rarely reported by our patients. One possible explanation for the discrepancy is that these symptoms are associated with thyroid dysfunction. Currently, routine thyrotropin (TSH) suppression is not recommended, and optimal TSH goals should balance the potential benefits of TSH suppression with the adverse effects from subclinical thyrotoxicosis^7^. Our patients mostly maintained low-normal serum TSH concentrations; therefore, the occurrence of these symptoms of hypothyroidism and hyperthyroidism was relatively infrequent.

Although some symptoms in the pharyngolaryngeal cluster of this study are not included in the MDASI-Thy questionnaire, these symptoms can be found in the MD Anderson Symptom Inventory for head and neck cancer (e.g. mucus in the mouth/throat, difficulty swallowing/chewing, choking or coughing, difficulty with voice/speech, problems with tasting food, and mouth/throat sores)^22^. Interestingly, the symptoms in the pharyngolaryngeal cluster were significantly more common in patients with disease recurrence than in patients without disease recurrence. The presence of multiple symptoms in a symptom cluster may represent possible common biological aetiologies. Hence, it might be related to a high frequency of locoregional recurrence observed in our series. Symptomatic recurrence has been adversely associated with survival in some cancer types^12,23^. Thus far, the prognostic impact of symptomatic versus asymptomatic recurrence in thyroid cancer remains unknown, and a prospective study is required to confirm the association between symptoms and outcomes after recurrence.

We also observed a high prevalence of symptoms in the psychoneurological cluster. A heightened level of fatigue has been noted in thyroid cancer survivors^24^. Moreover, the fatigue-pain cluster is a stronger predictor of overall health among advanced cancer patients^25^. In a recent longitudinal assessment of the quality of life, patients with low-risk papillary thyroid microcarcinoma who received active surveillance as primary management had significantly better physical and psychological health than those who had immediate surgery^26^. The finding illustrates the complexity of interactions between physical and psychological perspectives. It is probable that the symptoms in the pharyngolaryngeal cluster aggravate the symptoms in the psychoneurological cluster, although the two clusters showed a statistically non-significant but notable concordance.

Symptoms can be caused by the disease itself or by treatment-related side effects. The symptom burden exerts a significant impact on the quality of life and is highly prioritised by patients with thyroid cancer^27^. A problem-solving approach in the management of symptom problems is generally recommended^28^. Recently, an interdisciplinary team-based care approach has been shown to improve overall well-being and reduce physical and practical issues and concerns^29^. Based on our findings, we hypothesised that the symptom assessment may have a potential role to guide clinical surveillance. A proper symptom-driven evaluation in patients with thyroid cancer will not only tailor management to alleviate symptoms but also could serve as a ‘warning’ to signal recurrence.

It is generally accepted that familial thyroid cancer is more aggressive than any other cancers and possesses a higher recurrence rate^30^. Nonetheless, the influence of obesity on the clinicopathological features of thyroid cancer is controversial. In some studies, obesity was associated with advanced TNM stage or aggressive tumour features such as lymph node metastasis^15,31,32^. On the contrary, other studies suggested that obesity was not associated with aggressive tumour features or clinical outcomes^14,33^. The association between obesity and persistent or recurrent disease was not detected in most studies, probably because classical clinicopathological factors play a more important role, which leads to a type II error in smaller series. Consistent with the present observations, a large-scale study from France found that obese patients had an increased risk of developing locoregional events during the follow-up^34^. Accordingly, obese patients with thyroid cancer may be monitored more carefully for the early detection of persistent and recurrent disease.

The association between excessive weight and thyroid cancer includes insulin resistance, adipokines, and inflammation^35^. Adipokines are a set of cytokines secreted by the adipose tissue. The increase in the adipose tissue associated with obesity is associated with an increase in leptin levels and a decrease in adiponectin levels. Previously, we demonstrated that leptin and adiponectin receptors were expressed in a subset of thyroid cancer and provided prognostic information in addition to clinical characteristics^36–38^. Furthermore, adiposity is associated with a state of chronic inflammation. After the initial treatment of thyroid cancer, an increase in neutrophil-to-lymphocyte ratio suggests an incomplete response to therapy^39^. It is worth noting that bariatric surgery is associated with decreased risk of some hormone-related cancers^40^. Further studies on lifestyle intervention for weight management in obese patients with thyroid cancer are required.

Our study has some limitations. The retrospective design led to a non-uniform follow-up protocol and other inherent biases. Differences in symptom reporting are unnecessarily associated with the differences in symptom experiences. In this study, the symptoms were analysed as dichotomous variables. A Likert-type scale measuring the severity of the symptoms might increase the validity of the assessment. Furthermore, the changes in body weight during the follow-up were not taken into consideration. A longitudinal and more systematic investigation of the impact of symptom burden and body weight of patients with thyroid cancer is required. The analytical data comes from the dataset of a medical center which limiting the inference of research results.

In conclusion, using a matched-pair analysis to account for the potential confounders including age, sex, surgery, and TNM stage, we investigated a wide range of symptoms and demonstrated that patients with thyroid cancer with disease recurrence have higher subjective symptom burden, particularly in pharyngolaryngeal and psychoneurological clusters, than patients with thyroid cancer without disease recurrence. Family history of thyroid cancer and obesity are also associated with recurrence for the matched cohorts. These findings suggest that a careful attention should be paid to the possibility of recurrence for patients with high symptom distress in addition to symptomatic management.

## Data Availability

The datasets generated during and/or analysed during the current study are available from the corresponding author on a reasonable request.
